# A regression model for risk difference estimation in population-based case–control studies clarifies gender differences in lung cancer risk of smokers and never smokers

**DOI:** 10.1186/1471-2288-13-143

**Published:** 2013-11-19

**Authors:** Stephanie A Kovalchik, Sara De Matteis, Maria Teresa Landi, Neil E Caporaso, Ravi Varadhan, Dario Consonni, Andrew W Bergen, Hormuzd A Katki, Sholom Wacholder

**Affiliations:** 1 Economics, Sociology, and Statistics Department, RAND Corporation, Santa Monica, CA, USA; 2 Unit of Epidemiology, Department of Preventive Medicine, Fondazione IRCCS Ca’ Granda - Ospedale Maggiore Policlinico, Milan, Italy; 3 Division of Cancer Epidemiology and Genetics, National Cancer Institute, National Institutes of Health, Bethesda, MD, USA; 4 Division of Geriatric Medicine and Gerontology, Department of Medicine, Johns Hopkins University School of Medicine, Baltimore, MD, USA; 5 Molecular Genetics Program, Center for Health Sciences, SRI International, Menlo Park, CA, USA

**Keywords:** Additive risk, Absolute risk, Case–control study, EAGLE, Lung cancer, Risk assessment, Sex factors, Smoking

## Abstract

**Background:**

Additive risk models are necessary for understanding the joint effects of exposures on individual and population disease risk. Yet technical challenges have limited the consideration of additive risk models in case–control studies.

**Methods:**

Using a flexible risk regression model that allows additive and multiplicative components to estimate absolute risks and risk differences, we report a new analysis of data from the population-based case–control Environment And Genetics in Lung cancer Etiology study, conducted in Northern Italy between 2002–2005. The analysis provides estimates of the gender-specific absolute risk (cumulative risk) for non-smoking- and smoking-associated lung cancer, adjusted for demographic, occupational, and smoking history variables.

**Results:**

In the multiple-variable *lexpit* regression, the adjusted 3-year absolute risk of lung cancer in never smokers was 4.6 per 100,000 persons higher in women than men. However, the absolute increase in 3-year risk of lung cancer for every 10 additional pack-years smoked was less for women than men, 13.6 versus 52.9 per 100,000 persons.

**Conclusions:**

In a Northern Italian population, the absolute risk of lung cancer among never smokers is higher in women than men but among smokers is lower in women than men. *Lexpit* regression is a novel approach to additive-multiplicative risk modeling that can contribute to clearer interpretation of population-based case–control studies.

## Background

The multiplicative model quantifies the joint effects of exposures on the relative risk of disease and is the mainstay of case–control analysis [1]. The contribution of the multiplicative model to studies of disease etiology is undeniable. However, there are several epidemiological questions that are more easily addressed with an additive risk model, where exposure effects are modeled on the absolute risk (probability) scale. In particular, additive risk models can clarify the public health significance of exposure effects [2,3] and the interpretation of statistical interactions [4-6]. Despite these advantages, the technical difficulties of properly constraining risk estimates to the 0–1 range and a lack of software for constrained additive risk regression have hindered the use of additive risk models in case–control studies [7-9].

We recently encountered the challenge of additive risk modeling with case–control data in an investigation of gender differences in smoking-associated lung cancer in the Environment and Genetics in Lung cancer Etiology (EAGLE) Study—a population-based case–control study conducted in Northern Italy between 2002–2005 [10]. In a logistic regression analysis of never and ever smokers of the EAGLE Study, De Matteis and colleagues found evidence of an interaction between gender and pack-years smoked that suggested a higher susceptibility to lung cancer in men [11,12]. The authors sought to quantify the public health implications of the gender differences they found by estimating absolute risk differences of lung cancer in men and women, adjusted for other confounders. The risk difference estimates could theoretically be obtained with an additive risk model yet, unlike methods for multiplicative modeling, reliable methods for additive risk regression with case–control data were not available.

To address the challenge of absolute risk estimation in case–control studies, we present a novel regression approach to quantify risk difference associations with population-based case–control data using linear-expit (*lexpit*) regression. *Lexpit* regression is an additive-multiplicative risk model for a dichotomous outcome that can incorporate additive and multiplicative effects of risk factors and properly constrains risk estimates to a feasible range. We previously showed that *lexpit* regression addresses the main technical challenges to additive risk analysis of binary data in cohort studies [13]. Building on this earlier work, we extend *lexpit* regression to population-based case–control studies by incorporating sampling information into the estimation procedure. After describing the interpretation of *lexpit* regression and its methodology, we return to the question that motivated the development of these new methods and use the *lexpit* model to quantify confounder-adjusted risk difference effects of gender for smoking- and non-smoking associated lung cancer in the EAGLE Study.

## Methods

### Study participants

The EAGLE Study is a population-based case–control study of lung cancer in a Northern Italian population. Details of the study design have been previously described [10]. Briefly, during 34 months of observation between 2002–2005, 2,100 pathologically-confirmed cases of primary lung cancer were identified from 13 hospitals in the Lombardy region. A frequency-matched random sample of 2,120 controls was drawn from the Lombardy census using 90 strata defined by combinations of residence, age, and gender. Our analyses were based on the 1,943 cases (92.5%) and 2,116 controls (99.8%) who completed the study interview, and we assumed that the completion of an interview was non-informative for the risk association analyses. Table 1 shows the study’s sampling strata, the size of the control population, and the size of the control sample in each stratum. Approximate sampling fractions, the probability of a control subject’s selection, are equal to the control sample size divided by its population size. *Lexpit* analyses use the sampling information to estimate the association of selected exposures and the 3-year (rounded from 34 months) absolute risk of lung cancer. Since only a small percentage of cases and controls did not complete a study interview, population counts were derived from the interviewed sample without adjustment for interview completion.

**Table 1:** Projected control population by gender, age, and regional sampling strata in the EAGLE Study

	**Milan**	**Monza**	**Brescia**	**Pavia**	**Varese**
	***N Population (Number of controls)***				
Male					
35-39	70,630 (3)	12,352 (1)	22,479 (1)	9,202 (2)	9,866 (1)
40-44	58,166 (9)	10,045 (4)	18,816 (2)	7,958 (1)	8,156 (3)
45-49	50,727 (25)	9,143 (3)	16,644 (3)	7,046 (5)	7,698 (2)
50-54	55,952 (56)	9,677 (3)	17,760 (18)	7,508 (3)	8,155 (7)
55-59	51,407 (145)	8,281 (8)	14,665 (33)	5,951 (8)	6,783 (25)
60-64	55,106 (209)	9,083 (17)	14,682 (47)	6,765 (15)	7,127 (20)
65-69	45,477 (251)	7,043 (26)	11,334 (42)	5,855 (30)	5,765 (41)
70-74	35,965 (242)	5,423 (22)	8,995 (30)	4,518 (20)	4,640 (27)
75-80	24,960 (149)	3,430 (10)	6,291 (18)	3,417 (8)	3,278 (22)
Total	448,390 (1,089)	74,477 (94)	131,666 (194)	58,220 (92)	61,468 (148)
Female					
35-39	68,084 (5)	11,717 (1)	20,391 (1)	0 (0)	9,509 (2)
40-44	57,734 (2)	10,122 (1)	17,455 (1)	7,410 (4)	8,061 (1)
45-49	53,942 (13)	9,387 (4)	16,248 (7)	6,979 (3)	7,754 (6)
50-54	63,060 (27)	10,307 (1)	17,739 (3)	7,424 (7)	8,545 (2)
55-59	58,781 (61)	9,022 (2)	15,140 (7)	6,085 (5)	7,099 (6)
60-64	63,452 (43)	9,607 (5)	15,885 (8)	7,352 (4)	7,675 (3)
65-69	56,296 (73)	8,152 (5)	12,439 (9)	7,199 (7)	6,822 (4)
70-74	50,119 (63)	7,090 (3)	13,220 (7)	6,763 (4)	6,083 (4)
75-80	43,166 (62)	5,610 (1)	11,221 (10)	5,732 (3)	5,404 (9)
Total	514,634 (349)	81,014 (23)	139,738 (53)	49,212 (37)	66,952 (37)

Table 2 summarizes information on demographics, smoking history, and environmental smoking exposure in the cases and controls of the study sample, after excluding 157 cases and 4 controls without a complete baseline questionnaire. Men had greater smoking exposure than women overall, and in cases and controls separately. While 98% of male cases and 75% of male controls were current or former smokers, 75% of female cases and 43% of female controls had ever smoked. Compared to women of the same case status, men were more likely to have held a high-risk job [14], been exposed to tobacco smoke in the workplace, or to have smoked cigars, pipes, or cigarillos (Table 2).

**Table 2:** Descriptive characteristics by gender for the EAGLE study

	** Lung cancer cases (n=1,943)**		**Controls (n=2,116)**	
	**Male (n=1,537)**	**Female (n=406)**	**Male (n=1,617)**	**Female (n=499)**
Age				
35-59	301 (20)	123 (30)	371 (23)	172 (34)
60-66	428 (28)	91 (23)	469 (29)	104 (21)
67-71	362 (24)	77 (19)	366 (23)	94 (19)
72+	446 (28)	115 (28)	411 (25)	129 (26)
*P-value*		<0.001		<0.001
Education				
None	91 (6)	21 (5)	66 (4)	24 (5)
Elementary	625 (40)	128 (32)	431 (27)	143 (29)
Middle school	424 (28)	134 (33)	456 (28)	158 (31)
High school or more	397 (26)	123 (30)	664 (41)	174 (35)
*P-value*		0.005		0.0975
Ever had list A/B job				
Yes	522 (34)	27 (7)	447 (28)	28 (6)
No	1,015 (66)	379 (93)	1,170 (72)	471 (94)
*P-value*		<0.001		<0.001
ETS at the workplace				
Yes	1,180 (70)	215 (54)	1,127 (70)	270 (54)
No	357 (30)	191 (46)	490 (30)	229 (46)
*P-value*		<0.001		<0.001
***Smoking variables***				
Ever smoked cigars, pipes, or cigarillos				
Yes	267 (17)	5 (1)	309 (19)	2 (0)
No	1,270 (83)	401 (99)	1,308 (81)	497 (100)
*P-value*		<0.001		<0.001
Smoking status				
Never smoker	29 (2)	103 (25)	397 (25)	282 (57)
Former	723 (47)	116 (29)	800 (49)	110 (22)
Current	785 (51)	187 (46)	420 (26)	107 (21)
*P-value*		<0.001		<0.001
Pack-years (Smokers only)				
<5	38 (3)	31 (10)	265 (22)	97 (45)
5-19	82 (5)	56 (18)	199 (16)	50 (23)
20-39	421 (28)	124 (41)	413 (34)	49 (22)
40+	967 (64)	92 (31)	343 (28)	21 (10)
*P-value*		<0.001		<0.001
Years since quitting (Quitters only)				
<10	337 (52)	60 (47)	158 (20)	28 (25)
10+	386 (48)	56 (53)	642 (80)	82 (75)
*P-value*		0.3557		0.2059
Avg. percent inhaled (Smokers only)				
25	1 (0)	5 (2)	3 (0)	1 (0)
50	62 (4)	17 (6)	36 (3)	11 (5)
75	449 (30)	113 (37)	295 (24)	53 (24)
100	995 (66)	168 (55)	886 (73)	152 (70)
*P-value*		<0.001		0.3905

ETS = Environmental tobacco smoke.

P-values are based on a chi-squared test of gender differences by case status.

### *Lexpit* model

#### Description

The *lexpit* model relates risk factors ***x*** and ***z*** to the absolute risk (also called cumulative risk of disease or the probability of disease) over a fixed time interval. Here, both *x* and *z* can include multiple categorical or continuous variables. Denoting the absolute risk of disease within a fixed time τ given *x* and *z* as *R(x,z),* the *lexpit* model of cumulative risk is

(1) $$R\left(x,z\right)=\beta 'x+\exp\mathrm{it}\left(\gamma_{0}+\gamma 'z\right)$$

a sum of additive *β* ' *x* and multiplicative exp *it*(*γ*_0_ + *γ* ' *z*) components, where exp *it*(*u*) = *esp*(*u*)/(1 + exp(*u*)) is the inverse-logit (*expit*) function, which converts the log-odds *u* to the risk scale. An incidence rate can also be derived by dividing the cumulative risk *R(x,z)* by the length of the risk period *τ*, ⋅ *R*(*x*, *z*)/*τ*, under the assumption of constant risk over the risk period. For case–control study designs, the risk period length τ is typically equal to the duration of case ascertainment. When the additive terms of *lexpit* are set to zero, the model reduces to a strictly multiplicative logistic model; when the multiplicative terms are set to zero, the model reduces to a strictly additive binomial linear model.

The “additive” and “multiplicative” descriptions of the *lexpit* model coefficients refer to the effects of the ***x*** and *z* variables on the baseline risk, or the cumulative risk of disease in unexposed individuals, denoted as R_0_=expit(*γ*_*0*_*).* According to (1), the risk in a person with *x*=*x*_1_ exposure is *β* ' *x*_1_ greater than a person with *x*=*x*_1_-1; thus, each *β* coefficient is a risk difference associated with a unit increase in the corresponding ***x*** factor, after adjusting for all remaining *x* and *z* factors.

In Equation (1), the risk in a person with z=z_1_ is a *multiplicative* factor of the baseline risk approximately equal to ≈ exp(*γ* ' *z*_1_). The exponentiated value of each *γ* coefficient estimates the residual odds ratio associated with a unit increase in the corresponding *z* variable, after adjustment for the risk due to *x* exposures and the effects of remaining *z*. In logistic regression, coefficients are the adjusted log-odds ratios of odds having the form *R*(*x*, *z*)/(1 − *R*(*x*, *z*)). In *lexpit* regression, the log-odds ratios represented by the coefficients γ involve odds of the form (*R*(*x*, *x*) − *β* ' *x*)/(1 − *R*(*x*, *z*), where *R*(*x*, *z*) − *β* ' *x* is the risk that remains after subtracting the risk due to *x* exposures. Hence, we refer to the exponentiated coefficients γ of the *lexpit* model as “residual odds ratios”. These residual odds ratios are directly comparable to the odds ratio associations in a logistic regression model of *z* exposures fit to the subgroup of individuals without exposure to the *x* variables, i.e., with x = 0.

The baseline risk parameter *γ*_*0*_ is included in the *expit* for mathematical convenience, as no constraints are required to ensure that R_0_=expit(*γ*_*0*_*)* is within the feasible 0–1 probability range.

#### Example 1: Interpretation of lexpit model coefficients

To illustrate the interpretation of the coefficients of the *lexpit* model, suppose we perform a *lexpit* regression in the EAGLE Study to determine the risk difference in the 3-year risk of lung cancer associated with gender, controlling for the effect of smoking duration. The *lexpit* model in our example is

(2) $$R\left(x_{1},z_{1}\right)=\beta x_{1}+\exp\mathrm{it}\left(\gamma_{0}+\gamma z_{1}\right)$$

with univariate x_1_ for gender (1 = female, 0 = male) and univariate multiplicative term z_1_= Years Smoked, a continuous variable. Under model (2), the 3-year risk difference between a woman and a man with equal years smoked is *R*(1, *z*_1_) − (0, *z*_1_) = *β* Thus, *β* is the difference in lung cancer risk between women and men, adjusted for smoking.

Next we consider the independent effect of a 30-year smoking duration. Under model (2), the residual log-odds is lg *it*(*R*(3, 30)) = *γ*_0_ + 30*γ* for a man and log *it*(*R*(1, 30) − *β*) = *γ*_0_ + 30*γ* for a woman who have smoked for 30 years. For each, the difference in the residual log-odds compared to a never smoker (the log-odds ratio) is 30*γ*. Thus, *γ* represents increase in the odds of lung cancer associated with an additional year’s duration of smoking, adjusted for gender.

#### Estimation

*Application of lexpit* regression to population-based case–control data can generate absolute risk and risk difference estimates when an unbiased representation of the underlying population is available. As with expansion estimators in survey estimation [15], weighing each observation by its inverse sampling fraction, roughly, yields an estimate of the number of individuals representative of the “study base” [16]. To accommodate stratified sampling, we suppose the study base consists of *J* strata. Let *ij* index the *i*th individual within the *j*th stratum. The data vector for this individual is {*y*_*ij*_, *x*_*ij*_, *z*_*ij*_, *w*_*ij*_}, where *y*_*ij*_ indicates case status, ***x***_***ij***_ are additive risk factors, ***z***_***ij***_ are multiplicative risk factors, and *w*_*ij*_ is the sampling weight. For a population-based case–control study with complete case ascertainment and random sampling of controls within strata, the sample weights equal 1 for all cases and the ratio of the population size *N*_*j*_ to the number of sampled controls *n*_*j*_ (*N*_*j*_*/n*_*j*_) for controls in stratum *j*. The use of inverse probabilities as sampling weights allows our methodology to accommodate more complex case–control designs (frequency matching, individual matching, etc.).

We use constrained maximum likelihood methods to obtain estimates for the parameters of the *lexpit* model, maximizing the pseudo-log-likelihood

(3) $$\begin{array}{l} l\left(\beta ,\gamma_{0},\gamma \right)=\Sigma_{j}\Sigma_{i}w_{\mathrm{ij}}\\ \;\times \left[y_{\mathrm{ij}}\log\mathrm{it}\left(R\left(x_{\mathrm{ij}},z_{\mathrm{ij}}\right)\right)+\log\left(1-R\left(x_{\mathrm{ij}},z_{\mathrm{ij}}\right)\right)\right] \end{array}$$

If all sampling weights *w*_*ij*_ were equal to 1, as in a cohort design, Equation (3) would be the exact log-likelihood of a sample of Bernoulli random variables *y*_*ij*_*,* with expected event probabilities *E*[*y*_*ij*_ = 1] = *R*(*x*_*ij*_, *z*_*ij*_). The pseudo-likelihood approach extends the method proposed by Benichou and Wacholder [17] with additional constraints in the maximization to ensure that the estimated risks for all observed risk types are within the [0, 1] range. Estimates for *Θ* = (*β*, *γ*_0_, *γ*) are therefore the solutions to the following constrained optimization problem,

$$\hat{\Theta }=\arg\max_{\Theta }\left\{l\left(\Theta \right)\right\}\;\mathrm{and}\;\Theta \in F$$

where argmax_x_{*f*(*x*)} is the value of *x* where *f(x)* achieves its maximum value and *F* defines the feasible region for the parameter space,

$$F=\left\{0\leq \beta 'x+\exp\mathrm{it}\left(\gamma_{0}+\gamma 'z\right)\leq 1\right\}\;\mathrm{forall}\;\left(x,z\right)\in \left(X,Z\right)$$

The quantities ***X*** and ***Z*** refer to the complete set of risk factors in the study sample. The feasible region is constructed from the joint distribution of (***X, Z***), creating a separate constraint for each unique combination of observed ***x*** and **z** factors. The feasible region guarantees that the risk estimate for each observed exposure type is a population probability.

To impose the conditions of the feasible region, we have adapted a constrained optimization algorithm previously developed for cohort analyses [13]. *Lexpit* methods for case–control data are similar to regression methods for survey data. The use of sample weights makes the risk estimates of the *lexpit* model for case–control data; both require the same design considerations for accurate estimation of standard errors of estimates. We therefore use influence-based methods, a common approach for linearized variance estimation of survey statistics [18], to derive variances for the *lexpit* model’s risk estimates. In the Additional file 1 we summarize the optimization algorithm and the influence approach for obtaining variance estimates for the *lexpit* model parameters.

#### Example 2: Lexpit model estimation for case–control data

To illustrate the basic estimation concept, consider a *lexpit* model with a single additive effect for gender *x*_*ij*_ (1 = female, 0 = male), *R*(*x*_*ij*_, 0) = *βx*_*ij*_ = exp *it*(*γ*_0_). Table 1 indicates that 1,617 male controls were sampled from a population of 774,221; 499 female controls from a population of 851,550. Treating gender as the only stratification variable, the sampling weight for male controls was 774,221/1,61≈479 and for female controls was 851,550/499≈1,706. Given 1,537 total male cases and 406 total female cases of lung cancer during the 3-year ascertainment period, the weighted estimate for 3-year lung cancer risk in men is

$$\begin{array}{l} \exp\mathrm{it}\left({\hat{\gamma }}_{0}\right)=\frac{1,537}{1,537+\frac{774,221}{1,617}*1,617}\\ \;=\frac{1,537}{1,537+4,221}\approx 2.0/1,000 \end{array}$$

and for women

$$\begin{array}{l} \exp\mathrm{it}\left({\hat{\gamma }}_{0}\right)+\hat{\beta }\frac{406}{406+\frac{851,550}{499}*499}=\frac{406}{406+851,550}\\ \;\approx 0.5/1,000 \end{array}$$

A risk difference estimate of $\hat{\beta }=-1.5/1,000$ represents 0.15% lower risk for women than men This example conceptualizes how the sampling probabilities of a case–control study, when available, can be utilized to obtain population risk estimates.

#### Choice of additive and multiplicative effects

The flexibility of *lexpit* regression in allowing estimation of the effect of an exposure as additive, multiplicative, or (in some cases) both, can create uncertainty about an exposure’s true mode of effect. Although the *true* mode of effect can never be known, we provide three practical strategies to explore the functional form of a given risk-exposure relationship: a risk-exposure scatter plot that gives a graphical depiction between crude risk and a continuous exposure, a testing method based on the comparison of effects in a *lexpit* model with *both* additive and multiplicative effects of an exposure, and a measure of goodness-of-fit. Details of each approach are provided in the Additional file 1.

## Results

*Lexpit* regression was performed to assess the absolute risk differences associated with gender and smoking in the Northern Italian population represented by EAGLE participants. Our main interest was in a model that could estimate additive effects for gender, pack-years, and their interaction, considering multiplicative effects for all remaining covariates. A description of the included variables and their codings are described in Table 3. The *lexpit* analysis was conducted in the R language, version 2.15 [19], using our open-source package blm [20] (for usage examples see Additional file 2 and Additional file 3).

**Table 3:** Representation of variables included in regression analyses of the EAGLE study

**Factor**	**Representation**	**Values**
Pack-years^a^	Continuous	
Female	Categorical	Male = 0
		Female = 1
Age	Continuous	Years
Education	Trend	None = 0
		Elementary = 1
		Middle school = 2
		High school or more = 3
Smoked cigars, pipes, cigarillos	Categorical	Never Smoked = 0
		Smoked = 1
ETS in the workplace	Categorical	No ETS = 0
		ETS = 1
High-risk occupation^b^	Categorical	No occupation = 0
		Occupation = 1
Average percent inhaled	Trend	Never smoker = 0
		<25% = 1
		25-49% = 2
		50-74% = 3
		75-100% = 4
Years since quitting	Continuous	Years

ETS = Environmental tobacco smoke.

^a^ Average packs of cigarettes smoked per day x years smoked.

^b^ List A/B high-risk occupation for lung cancer.

Estimates for the additive effects of gender showed a 4.6 per 100,000 persons higher 3-year lung cancer risk for women than men among never smokers, adjusting for other demographic variables (Table 4). The risk difference effect can also be expressed as a rate by dividing by the duration of risk, e.g. a 4.6 per 100,000 34-month risk corresponds to an average risk rate of 13 per 100,000 person-years. We estimate that every 10 additional pack-years smoked increases the 3-year lung cancer risk in male smokers by 52.9 per 100,000 persons but by only 13.6 per 100,000 persons among women, showing a strong female-pack-year interaction (RD=−39.3 per 100,000 persons per 10 pack-years smoked, 95% CI=−70.1 to −8.6). After accounting for the risk effects of gender and pack-years, the residual odds ratio effects of the *lexpit* model found that greater age, occupational ETS exposure, and inhalation depth further increased lung cancer risk estimates, while higher education and greater years since quitting decreased risk estimates.

**Table 4:** ***Lexpit *** **regression analysis of the EAGLE Study**

**Factor**	**3-year risk difference (per 100,000)**	**95% confidence interval**	**Residual odds ratio**	**95% confidence interval**
Female	4.6	(−1.8, 11.0)		
Pack-years (per 10 yrs)	52.9	(31.9, 73.8)		
Female x Pack-years	−39.3	(−70.1, -8.6)		
Age – 60^a^			1.12	(1.10, 1.13)
Education – 1^b^			0.69	(0.60, 0.80)
High-risk occupation^c^			1.01	(0.72, 1.41)
Occupational ETS			1.54	(0.72, 1.41)
Cigars, pipes, cigarillos			1.15	(0.86, 1.53)
Average percent inhaled			2.19	(1.99, 2.41)
Years since quitting			0.94	(0.93, 0.95)

ETS = Environmental tobacco smoke.

^a^ Centered at 60 years.

^b^ Centered at 1, corresponding to elementary school.

^c^ List A/B high-risk occupation for lung cancer.

As one assessment of the improvement of the fit of the model with the use of multiplicative effects we compared the weighted Hosmer-Lemeshow goodness-of-fit statistic (Additional file 1: Section S3) among the *lexpit* model, a strictly additive blm model, and a strictly multiplicative logistic model of the same variables. The chi-squared statistic in the blm model was 20.8, the weighted logistic model 18.2, and 15.9 with the *lexpit* model, indicating an improvement in fit with the use of the additive-multiplicative form we used.

## Discussion

We have presented *lexpit* regression methods to estimate adjusted absolute risk differences with population-based case–control data. By shifting the focus from estimates of relative risk to absolute risk, *lexpit* regression gives epidemiologists a direct and reliable way to assess the public health significance of an exposure’s effect. Moreover, *lexpit* regression provides a flexible framework for handling potential confounders, as variables with additive or multiplicative effects can be accommodated. When there is uncertainty about a variable’s mode of effect, we outlined approaches to assess the reasonableness of each effect type. Our open-source R package blm allows the new methods to be implemented with the ease of standard logistic regression.

*Lexpit* regression is the absolute risk analog to additive-multiplicative models for hazard rates, such as the Cox-Aalen model [21], which have become increasingly popular in the survival literature [22]. Each class of models share the strength of greater flexibility in the study and representation of the joint effects of risk factors on the hazard rate, in the case of the Cox-Aalen model, and the absolute risk of disease, in the case of the *lexpit* model. The extension of additive-multiplicative models to absolute risk estimation from a variety of study designs is significant because of the importance of individualized risk assessment to public health. To our knowledge, the *lexpit* model is the first additive-multiplicative regression model of risk that appropriately ensures risk estimates are within the probability scale. Although alternative additive-multiplicative models of risk could be developed by considering other functions for the multiplicative component (e.g. exp), we have focused on the *expit* function because of its mathematical advantages. Because of the expit function, the *lexpit* model will require fewer constraints than alternative additive-multiplicative models to produce feasible estimates in the 0–1 probability range.

None of more than 20 published observational studies that have examined male–female differences in lung cancer etiology have quantified the independent effect of gender on the absolute risk of smoking- and non-smoking-associated lung cancer [23-26]. Using *lexpit* regression, we were able to address this important public health question. Our findings add to the De Matteis *et al.* logistic regression of the EAGLE case–control study [11] in two important ways. First, we confirmed that gender differences in the confounder-adjusted effect of pack-years are found on the additive risk scale. Secondly, we found suggestive evidence that women’s risk of lung cancer risk is higher than men’s in never smokers but is lower than men’s in smokers. Conventional unconditional logistic regression, which does not provide estimates of absolute risk, would not identify these findings, especially given that gender was used as a matching variable in selecting controls. Thus, our novel methods provide further insight about male-and-female differences in lung cancer risk from previously analyzed data that has direct public health implications.

In their commentary on the De Matteis *et al*. study, Alberg and colleagues pointed to a need to further delineate the clinical significance of gender differences in lung cancer etiology [12]. Our re-analysis of the EAGLE Study clarifies the clinical relevance of gender effects for lung cancer risk in an Italian population by providing estimates of the excess lung cancer risk associated with gender. The small excess risk in women never smokers suggests that some gender-related etiological factor(s) for non-smoking-related lung cancer remains to be identified. A public health implication for the gender differences we found among smokers concerns selection criteria for computed tomographic lung cancer screening. Current guidelines recommend screening for individuals between ages 55 and 75 years with a minimum of 30 pack-years smoked [27]. However, in an Italian population, we estimate that the excess lung cancer risk for a male 30 pack-year smoker is more than 1,100 per 100,000 greater than an otherwise similar female 30 pack-year smoker. Thus, in keeping with the “equal management for equal risk” principle [28], gender-based risk criteria for lung cancer screening selection may be warranted in some populations.

The implications of the EAGLE *lexpit* analysis for computed tomographic screening guidelines exemplifies the importance of the choice of measure of association used in an etiological analysis for understanding the public health significance of a risk factor’s effect. Risk differences measure a risk factor’s effect in terms of the number of excess attributable cases in a well-defined population, an explicit measure of the public health significance of an effect, which can be compared across exposures and across diseases. Our study provides an important example of this comparative use of risk differences with respect to gender effects in smoking- and non-smoking-associated lung cancer. Some research has suggested a higher risk of lung cancer among women never smokers [29]. We further elucidated this difference through *lexpit* analysis by showing that the excess risk in women never smokers was approximately equal to the excess risk with 1 additional pack-year smoked in men as compared to women. As the development of public health interventions and clinical recommendations become increasingly guided by individual risk assessment, there will be a growing need for methods like *lexpit* regression that can facilitate the estimation of absolute risk differences from observational data.

*Lexpit* regression resolves several limitations of alternative strategies for estimating risk differences from case–control studies. Using non-additive models of risk, such as the logistic model, to estimate a marginal risk difference [30,31] gives average in the study population, not equivalent to a risk difference effect estimated here. The application of the *lexpit* model to case–control data extends previously proposed methods for absolute risk methods requiring prospective cohorts or disease registries [32]. Further, *lexpit* regression advances current methods for assessing additive interactions in case–control studies. It is well known that multiplicative interactions sometimes disappear when modeled on the additive scale [4-6,33] and vice versa, highlighting the dependence of statistical interactions on the choice of a model’s scale. The removal of interactions leads to more parsimonious models whose risk associations have a clearer interpretation. The flexible additive-multiplicative form of the *lexpit* can help epidemiologists reduce multiplicative and additive statistical interactions, making it easier to interpret risk effects. While departure from additivity can be detected on the relative risk scale using the relative excess risk due to interaction, this metric is limited because it can only detect the direction of departure from additivity but not the magnitude of the effect [34,35].

While *lexpit* regression makes the important advance of allowing case–control studies to make inferences about absolute risk and risk differences of exposures, there are several challenges to its application to case–control data. First, the period of risk for the cumulative risk estimates of the *lexpit* model is determined by the period of case ascertainment, which may generally prohibit long-term risk estimates. As with other common probability models of case–control data, the *lexpit* model assumes the population risk of disease is fixed during the period cases and controls are sampled. The population validity of *lexpit* regression also requires accurate sampling weights, which may be difficult to obtain for studies using a so-called “secondary base” [36], as with hospital or registry controls, for the selection of controls. Further investigation of the availability and accuracy of sampling information in case–control studies is needed to clarify the practical limitations of using sampling data for absolute risk estimation.

## Conclusions

Additive and multiplicative models concern “two quite different aspects of the association between risk factor and disease” [1], p. 58. Epidemiologists have been urged to consider both perspectives in risk association studies, especially in the assessment of effect modification [26], yet technical challenges have long made multiplicative models more convenient to use. In this paper, we have presented methods and software [27] to allow analyses of population-based case–control studies to incorporate these complementary perspectives into a single model via *lexpit* regression. Further applications and extensions of additive risk modeling with case–control data will help to improve our understanding of the joint effects of exposures on disease risk.

## Abbreviations

EAGLE: Environment and genetics in lung cancer etiology; Lexpit: Linear-expit.

## Competing interests

The authors have no competing interests to declare.

## Authors’ contributions

SAK designed and conducted the study analyses and wrote the first draft of the manuscript. SW, NEC, and MTL conceived of the design of the study sample and the coordination of data collection. SW, HAK, SDM, DC, AWB, and RV provided input on the statistical analyses and the interpretation of the results. All authors contributed to the writing of the manuscript and read and approved the final manuscript.

## Pre-publication history

The pre-publication history for this paper can be accessed here:

http://www.biomedcentral.com/1471-2288/13/143/prepub

## Supplementary Material

Additional file 1 Supplementary technical appendix. Click here for file

Additional file 2 R script file showing examples of lexpit modeling and supporting functions using the R blm package. Click here for file

Additional file 3 **Simulated population-based case–control dataset modeled after the EAGLE study design.** The examples presented in example.R make use of this dataset. Click here for file
