# Supplementary material for: A regression model for risk difference estimation in population-based case–control studies clarifies gender differences in lung cancer risk of smokers and never smokers
Source: BMC Med Res Methodol. 2013 Nov 19;13:143. doi: 10.1186/1471-2288-13-143 (PMC3840559; doi:10.1186/1471-2288-13-143)
Supplement: Additional file 1 — Supplementary technical appendix. [file 1471-2288-13-143-S1.docx]

**Supplementary Appendix to**

“A regression model for risk difference estimation in population-based case-control studies clarifies gender differences in lung cancer risk of smokers and never smokers”

*S1. Optimization algorithm*

We use an iterative two-stage approach to maximize the deviance of the *lexpit* model while satisfying the constraint that every fitted probability lies between zero and one. In Stage 1, *expit* terms are considered fixed and the pseudo-log-likelihood is maximized with respect to$\beta$ using an adaptive barrier algorithm with risk offset [1]. In Stage 2, the linear terms are treated as fixed and an iterative reweighted least squares algorithm with risk offset is used to maximize the pseudo-log-likelihood with respect to *γ*. For simplicity, in what follows we include the intercept term $\gamma_{0}$in $\gamma$.

*Stage 1: Linear terms*

Let for the current iteration. We regard *q_ij_* as a fixed offset in $R(x_{ij},z_{ij})$, *.* Optimization at the first stage maximizes $l(\beta)$,

$$l(\beta)=\sum_{j} \sum_{i} w_{ij}[y_{ij}\text{logit}(\beta'x_{ij}+q_{ij})+\log(1-\beta'x_{ij}-q_{ij})]$$

subject to the constraints of the feasible region *F*

$$F=\{-q_{ij}\leq\beta'x_{ij}\leq1-q_{ij}\}$$

for all *i* and *j*.

*Stage 2: Expit terms*

Let , where$\hat{\beta}$ is the update estimates from Stage 1. We regard *p_ij_* as fixed and optimize the pseudo-log-likelihood with respect to $\gamma$ using standard iterative reweighted least squares with offset *p_ij_*. The objective function is

$l(\gamma)=\sum_{j} \sum_{i} w_{ij}[y_{ij}\text{logit}(p_{ij}+\text{expit}(\gamma'z_{ij}))+\log(1-p_{ij}-\text{expit}(\gamma'z_{ij}))]$.

The algorithm iterates between Stages 1 and 2 until convergence. Convergence of the overall algorithm is guaranteed when the weighted-likelihood increases monotonically at each stage.

*Initialization*

To initialize the algorithm, the baseline rate of the model is set to

$\text{expit}(\hat{\gamma}_{0})=\sum_{j} \sum_{i} w_{ij}y_{ij}/\sum_{j} \sum_{i} w_{ij}$.

Other parameters are initialized at zero.

*S2. Inference*

Variances for$\hat{\beta}$ and $\hat{\gamma}$ are estimated using an influence-based method [2]. The sample influence operator is an estimate of the Gâteaux derivative of a functional [3], which, in *lexpit* analysis, is a regression parameter. The influence operator applied to a given data point estimates how a functional is changed by the addition of that data point. Thus, the analytic estimate of a jackknife residual provided by the influence operator can assess robustness [4] and simplify derivation of variances of estimators [5]. The estimate of the influence of the *ij*th individual on $\hat{\beta}$ is

and for $\hat{\gamma}$ is

In these expressions$H(\theta)$ denotes the Hessian matrix of $\theta$--the second partial derivative of the pseudo log-likelihood with respect to $\theta$.

Given the influence measures, the variance estimates for the model coefficients are

and

,

using to denote the mean of the influence measures within the *j*th stratum. In unmatched case-control studies, there are two strata, cases and controls. For frequency-matched case-control studies with *J* strata based on matching variables, the number of strata for the variance calculation is *2J*, as case status is treated as an additional level of stratification. The approaches we have outlined can be easily extended to more complex sampling designs [5].

*S3. Choice of Additive and Multiplicative Effects*

1. *Risk-exposure scatter plot*. We have created the risk-exposure scatter plot to reveal the relationship between a continuous exposure *x* (e.g. age, pack-years, etc.) and risk. This graphical method is conceptually similar to the Subpopulation Treatment Effect Pattern Plot [] but describes a continuous covariate’s relationship with risk (a one-sample description) rather than a treatment effect (a two-sample description). Risk estimates are computed for overlapping groups of 20% of the study sample. Groups are formed according to exposure status, beginning with the least exposed and forming new groups by sequentially adding the next 1% of persons with greater exposure. To formalize this process, let *Q(k*) be the observed value of the *x* exposure at which *k%* of observations have an exposure ≤*Q(k).* Define $\bar{x}(k)$ as the mean exposure value for the 20% of the study sample with the highest exposure values ≤*Q(k),*

$\bar{x}\left( k \right)={\sum_{j} \sum_{i} I\left( Q(k-20)\leq x_{\mathrm{ij}}\leq Q(k \right))w_{\mathrm{ij}}x_{\mathrm{ij}}}/{\sum_{j} \sum_{i} I\left( Q(k-20)\leq x_{\mathrm{ij}}\leq Q(k \right))w_{\mathrm{ij}}}$where *I(C)* is an indicator function that takes the value 1 if condition *C*  is met and 0 otherwise. Let $\bar{r}(k)$ be the corresponding crude risk in the same subgroup,

$\bar{r}\left( k \right)={\sum_{j} \sum_{i} I\left( Q(k-20)\leq x_{\mathrm{ij}}\leq Q(k \right))w_{\mathrm{ij}}y_{\mathrm{ij}}}/{\sum_{j} \sum_{i} I\left( Q(k-20)\leq x_{\mathrm{ij}}\leq Q(k \right))w_{\mathrm{ij}}}$.

To *see* the observed relationship between crude risk and the exposure *x*, we plot $\bar{r}(k)$ versus $\bar{x}(k)$ for *k=20,…,100*. The reasonableness of an additive effect due to *x* is indicated by the linearity of the scatter plot.

We used the risk-exposure plot to assess the reasonableness of the linearity assumption for pack-years in the *lexpit* analysis in EAGLE. Figure S1 indicates a linear relationship between unadjusted lung cancer risk and pack-years smoked in women smokers. For male smokers, the linearity assumption appears most suitable when the level of exposure is ≥20 pack-years. Since the majority of male smokers in EAGLE reported a number of pack-years within this range, we decided to perform the *lexpit* analysis with continuous pack-years as an additive term.

2. *Testing both additive and multiplicative marginal effects of a variable*. When the *x* exposure is not the only variable in the model, additive and multiplicative effects of *x* can both be included because these terms will not be collinear. When both additive and multiplicative effects are modeled, the significance of each effect (based on a Score test, for example) is an indication of its strength independent of the alternative mode of effect.

*3. Goodness-of-fit.* An indirect measure of the appropriateness of a specified exposure in a *lexpit* regression analysis is the overall fit of the model. A population-based Hosmer-Lemeshow goodness-of-fit statistic can be constructed by calculating the squared deviations of observed and expected cases and controls by the deciles of risk [7]. Let *M_ij_(k)* be an indicator of whether the *ijth* subject’s predicted risk is within the *kth* decile. The sum of squared deviances for controls is

$$X_{0}^{2}=\sum_{k} \frac{[\sum_{j} \sum_{i} M_{ij}(k)w_{ij}(y_{ij}-R(x_{ij},z_{ij}))^{2}]}{\sum_{j} \sum_{i} M_{ij}(k)w_{ij}(1-R(x_{ij},z_{ij}))}$$

and cases is

$$X_{1}^{2}=\sum_{k} \frac{[\sum_{j} \sum_{i} M_{ij}(k)w_{ij}(y_{ij}-R(x_{ij},z_{ij}))^{2}]}{\sum_{j} \sum_{i} M_{ij}(k)w_{ij}R(x_{ij},z_{ij})}.$$

The sum ${X^{2}=X}_{0}^{2}+X_{1}^{2}$ is the goodness-of-fit statistic. Larger values of *X^2^* indicate a greater lack of fit. The significance of the lack of fit can be tested by comparing *X^2^* to a chi-squared distribution with 8 degrees of freedom.

References

Figure S1. Risk-exposure scatter plot of 3-year cumulative lung cancer risk against pack-years smoked. Each point is based on 20% of the gender-specific subsample. The vertical grey line denotes the average pack-years smoked among the 20% of females with the greatest number of pack-years. This line highlights the limited information about the relationship between pack-years smoked and risk in females for levels of exposure >40 pack-years.

1. Lange K. *Numerical Analysis for Statisticians.* Springer, New York; 2010.
2. Deville J. Variance estimation for complex statistics and estimators: linearization and residual techniques. *Surv Methodol*. 1999;25:193–204.
3. Serfling RJ. Generalized L-, M-, and R-statistics. *Ann Stat.* 1984;12(1):76-86.
4. Hampel FR. The influence curve and its role in robust estimation. *J Am Stat Assoc.* 1974;69: 383–394.
5. Graubard BI, Fears TR. Standard errors for attributable risk for simple and complex sample designs. *Biometrics*. 2005;61(3):847–855.
6. Lazar AA, Cole BF, Bonetti M, Gelber RD. Evaluation of treatment-effect heterogeneity using biomarkers measured on a continuous scale: Subpopulation Treatment Effect Pattern Plot. *J Clin Oncol*. 2010;28(29):4539-4544.
7. Archer KJ, Lemeshow S. Goodness-of-fit test for a logistic regression model fitted using survey sample data. *Stata Journal*. 2006;6:97—105.
